# Individualized genetic network analysis reveals new therapeutic vulnerabilities in 6,700 cancer genomes

**DOI:** 10.1371/journal.pcbi.1007701

**Published:** 2020-02-26

**Authors:** Chuang Liu, Junfei Zhao, Weiqiang Lu, Yao Dai, Jennifer Hockings, Yadi Zhou, Ruth Nussinov, Charis Eng, Feixiong Cheng

**Affiliations:** 1 Alibaba Research Center for Complexity Sciences, Hangzhou Normal University, Hangzhou, Zhejiang, China; 2 Department of Systems Biology, Columbia University, New York, New York, United States of America; 3 Department of Biomedical Informatics, Columbia University, New York, New York, United States of America; 4 Shanghai Key Laboratory of Regulatory Biology, Institute of Biomedical Sciences and School of Life Sciences, East China Normal University, Shanghai, China; 5 Genomic Medicine Institute, Lerner Research Institute, Cleveland Clinic, Cleveland, Ohio, United States of America; 6 Cancer and Inflammation Program, Leidos Biomedical Research, Inc., Frederick National Laboratory for Cancer Research, National Cancer Institute at Frederick, Frederick, Maryland, United States of America; 7 Department of Human Molecular Genetics and Biochemistry, Sackler School of Medicine, Tel Aviv University, Tel Aviv, Israel; 8 Department of Molecular Medicine, Cleveland Clinic Lerner College of Medicine, Case Western Reserve University, Cleveland, Ohio, United States of America; 9 Case Comprehensive Cancer Center, Case Western Reserve University School of Medicine, Cleveland, Ohio, United States of America; 10 Taussig Cancer Institute, Cleveland Clinic, Cleveland, Ohio, United States of America; 11 Department of Genetics and Genome Sciences, Case Western Reserve University School of Medicine, Cleveland, Ohio, United States of America; University of California Irvine, UNITED STATES

## Abstract

Tumor-specific genomic alterations allow systematic identification of genetic interactions that promote tumorigenesis and tumor vulnerabilities, offering novel strategies for development of targeted therapies for individual patients. We develop an Individualized Network-based Co-Mutation (INCM) methodology by inspecting over 2.5 million nonsynonymous somatic mutations derived from 6,789 tumor exomes across 14 cancer types from The Cancer Genome Atlas. Our INCM analysis reveals a higher genetic interaction burden on the significantly mutated genes, experimentally validated cancer genes, chromosome regulatory factors, and DNA damage repair genes, as compared to human pan-cancer essential genes identified by CRISPR-Cas9 screenings on 324 cancer cell lines. We find that genes involved in the cancer type-specific genetic subnetworks identified by INCM are significantly enriched in established cancer pathways, and the INCM-inferred putative genetic interactions are correlated with patient survival. By analyzing drug pharmacogenomics profiles from the Genomics of Drug Sensitivity in Cancer database, we show that the network-predicted putative genetic interactions (e.g., BRCA2-TP53) are significantly correlated with sensitivity/resistance of multiple therapeutic agents. We experimentally validated that afatinib has the strongest cytotoxic activity on BT474 (IC_50_ = 55.5 nM, *BRCA2* and *TP53* co-mutant) compared to MCF7 (IC_50_ = 7.7 μM, both *BRCA2* and *TP53* wild type) and MDA-MB-231 (IC_50_ = 7.9 μM, *BRCA2* wild type but *TP53* mutant). Finally, drug-target network analysis reveals several potential druggable genetic interactions by targeting tumor vulnerabilities. This study offers a powerful network-based methodology for identification of candidate therapeutic pathways that target tumor vulnerabilities and prioritization of potential pharmacogenomics biomarkers for development of personalized cancer medicine.

## Author summary

Recent efforts to map genetic interactions in tumor cells have suggested that tumor vulnerabilities can be exploited for development of novel targeted therapies. Tumor-specific genomic alterations derived from multi-center cancer genome projects allow identification of genetic interactions that promote tumor vulnerabilities, offering novel strategies for development of targeted cancer therapies. This study develops a novel Individualized Network-based Co-Mutation (termed INCM) methodology for quantifying the putative genetic interactions in cancer. Trained on over 2.5 million nonsynonymous somatic mutations derived from 6,789 tumor exomes across 14 cancer type, we found that genes identified in the cancer type-specific genetic subnetworks were significantly enriched in established cancer pathways. The network-predicted putative genetic interactions are correlated with patient survival. By analyzing drug pharmacogenomics profiles, we showed that the network-predicted putative genetic interactions (e.g., BRCA2-TP53) were significantly correlated with sensitivity/resistance of anticancer drugs (e.g., afatinib) and we experimentally validated it in breast cancer cell lines. Finally, drug-target network analysis reveals several potential druggable genetic interactions (e.g., PIK3CA-PTEN) by targeting tumor vulnerabilities. This study offers a generalizable network-based approach for comprehensive identification of candidate therapeutic pathways that target tumor vulnerabilities and prioritization of potential prognostic and pharmacogenomics biomarkers for development of personalized cancer medicine.

## Introduction

Recent exponential advances in genome sequencing technologies have enabled a detailed map of genomic alterations identified in human cancer populations. Several multi-center cancer exome/genome projects, such as The Cancer Genome Atlas (TCGA) and the International Cancer Genome Consortium (ICGC), have significantly improved our understanding of the landscape of somatic alterations that promote tumorigenesis and tumor evolution [1–4]. Yet, the annual number of innovative anticancer agents approved by the U.S. Food and Drug Administration (FDA) has not increased significantly in the past few years compared to one or two decades ago [5]. There is a pressing need to develop new technologies, such as computational tools, to accelerate the modern oncology drug discovery and development by exploiting the wealth of large-scale exome/genome sequencing data in the genomics era from the evolutionary medicine perspective [6].

Somatic alterations identified in tumor exomes/genomes are commonly grouped into two classes: gain-of-function mutations on oncogenes and loss-of-function mutations on tumor suppressor genes (TSGs). Although inhibiting proteins encoded by oncogenes with small molecules or monoclonal antibodies have been proven to be effective in the clinic, it is challenging to inhibit the function of multiple undruggable oncogenes (i.e., *KRAS* and c-*Myc*) and the emerging drug resistance. In addition, restoring the function of TSGs that are highly mutated or deleted in tumor cells by traditional small molecular drug is not feasible. Recent efforts to map genetic interactions (e.g., synthetic lethal interactions) in tumor cells have suggested that tumor vulnerabilities from evolutionary medicine perspective can be exploited for development of novel molecularly targeted therapies [7–14].

In the context of genetic studies, a synthetic lethal interaction involves two genes: the cell is viable upon perturbation of either gene alone, but simultaneous perturbation of both genes by genetic or genomic alterations will result in cell death [9]. A synthetic lethal interaction occurring between a tumor-specific somatic mutation and a gene that drives tumorigenesis and tumor progression offers an ideal therapeutic target in cancer [10]. Furthermore, discovery of synthetic lethal interactions through identification of a second-site synthetic lethal druggable target facilitates indirect targeting of tumor alterations of undruggable proteins (e.g., KRas or p53) [7–9]. Recent advances in functional genomic technologies, such as RNA interference (RNAi) or CRISPR-Cas9 assays, have offered innovative tools to screen human cancer cells for genetic interactions. By systematic application of CRISPR-based screens, Wang et al., uncovered PREX1, a key synthetic lethal interactor of oncogenic Ras in human acute myeloid leukemia cell lines [8]. However, measurements of cell proliferation in genome-scale CRISPR-Cas9 loss-of-function screens have a potentially high false-positive rate in copy number-amplified regions [15, 16]. Furthermore, large-scale experimental assays are expensive and time-consuming. Computational approaches with low cost and high efficiency offer new tools for genome-wide identification of cancer genetic interactions and for inferring tumor evolution through analyzing publicly available large-scale tumor exome/genome sequencing data [17, 18]. For example, several computational approaches, such as MEMo [19] and WeSME [20], were reported to identify mutually exclusive mutations in cancer.

In this study, we propose a novel computational methodology, termed Individualized Network-based Co-Mutation (INCM), for comprehensive identification of putative genetic interactions that drive tumorigenesis and anticancer drug responses. The central hypothesis asserts that network-based co-mutation analysis of individual tumors may identify putative genetic interactions that promote tumorigenesis and tumor evolution, offering potential targets for the development of molecularly targeted therapies. Specifically, we applied INCM to over 2.5 million nonsynonymous somatic mutations derived from 6,789 tumor exomes across 14 cancer types from TCGA. We computationally identified hundreds of new putative genetic interactions in multiple cancer types via INCM. As proof-of-concept, we showed a higher genetic interaction burden mediated by significantly mutated genes in cancer populations, experimentally validated cancer genes, chromosome regulation factors, and DNA damage repair genes, in comparison to pan-cancer essential genes identified by CRISPR-Cas9 screenings in 324 cancer cell lines across 30 cancer types. Via INCM, we constructed cancer type-specific genetic interaction subnetworks for 14 cancer types respectively. We showed that network-predicted putative genetic interactions offer potential therapeutic targets and can be used to predict patient survival and drug responses. Put together, this study offers a generalizable network-based framework in identifying potential therapeutic pathways for personalized cancer medicine by targeting tumor vulnerabilities.

## Results

### Network-based Co-mutation analysis across individual tumors

**Fig 1:**
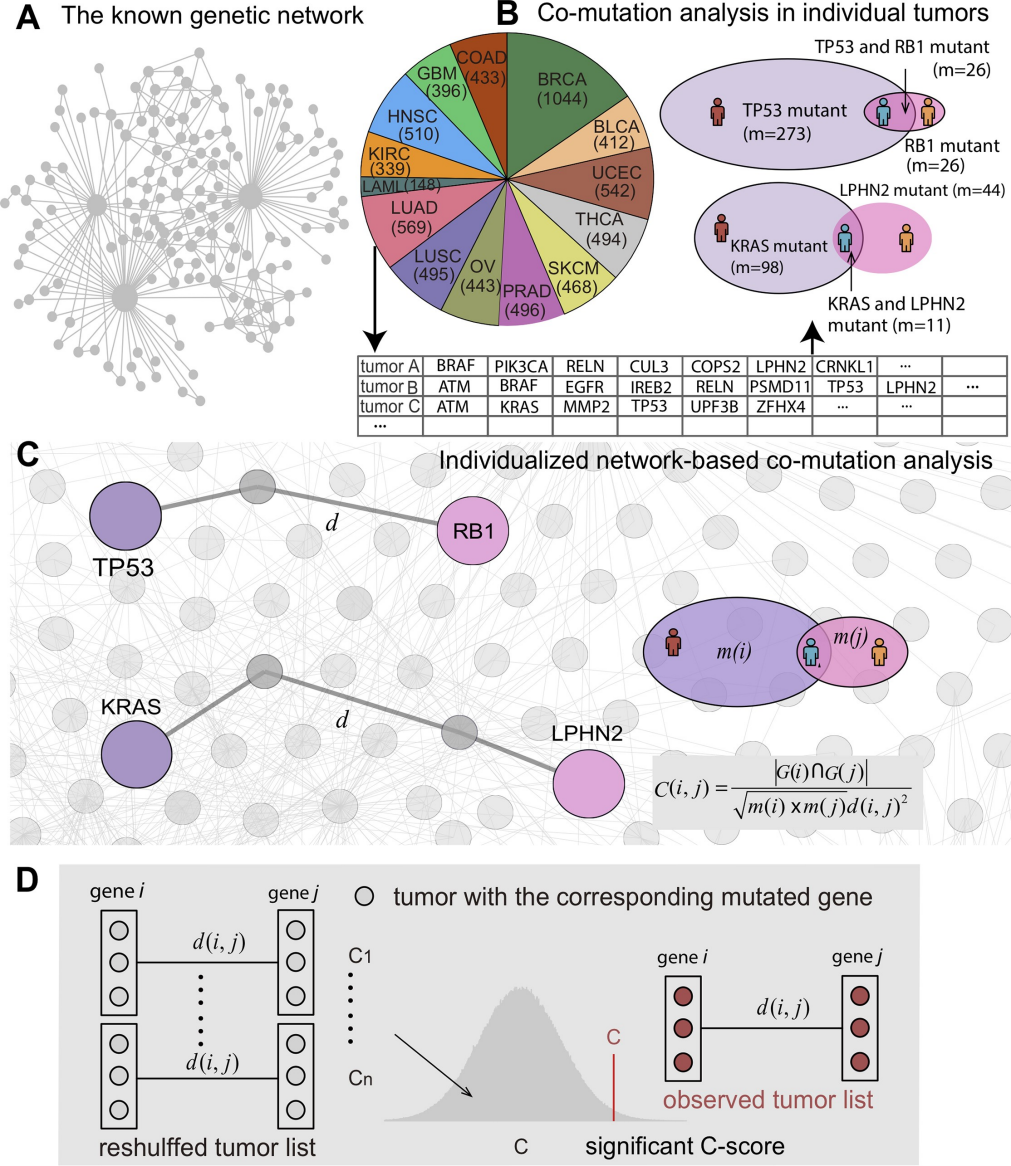
Diagram illustrating the pipeline of Individualized Network-based Co-Mutation (INCM) measure. (**A**) A network diagram showing prior topological architecture in the known human genetic interaction network. (**B**) Network-based co-mutation analysis by integrating over 2.5 million nonsynonymous somatic mutations derived from 6,789 tumor exomes across 14 cancer types from TCGA. (**C**) Individualized network-based co-mutation measure. The *d* denotes the shortest path length between genes *i* and *j* in the cancer type-specific co-expressed human genetic interaction network (see Methods). (**D**) The INCM measure (C-score) integrates the somatic mutations and network topology information of mutated genes in the experimentally validated human genetic interaction network (see Methods).

Previous studies have shown that gene pairs with high co-mutation rate in cancer populations and with the closest network topological distance in the human protein-protein interactome can have high likelihood to promote tumorigenesis and anticancer drug responses [21, 22]. In this study, we developed an INCM measure for quantifying putative genetic interactions in pan-cancer analysis. We calculated the INCM value (*C-Score*) by integrating over 2.5 million nonsynonymous somatic mutations derived from 6,789 tumor exomes across 14 cancer type from TCGA (**S1 Table**) into experimentally confirmed, cancer type-specific co-expressed genetic interaction network (**S2 Table**) under the prior network topological architecture (**Fig 1A**) as below:

$$C(i,j)=\frac{\left|G(i)\cap G(j)\right|}{\sqrt{m(i)\times m(j)}{d(i,j)}^{2}}$$ (1)

where |*G*(*i*)∩*G*(*j*)| represents the number of individual tumors where both genes *i* and *j* are the mutated genes (the number of the tumor overlap of *G*(*i*) and *G*(*j*)), *m*(*i*) and *m*(*j*) are the cumulative mutations of genes *i* and *j* respectively, and *d*(*i*,*j*) is the shortest path length between genes *i* and *j* in the cancer type-specific co-expressed human genetic interaction network. The INCM measure (*C-Score*) combines the somatic mutations and network topology information of mutated genes in the known human genetic interaction network. The somatic mutations-based correlation ($\frac{\left|G(i)\cap G(j)\right|}{\sqrt{m(i)\times m(j)}}$), which is referred to as the cosine similarity, indicates the co-mutation probability in individual tumors between gene-pairs of two co-mutated genes based on the network hypothesis described in our previous study [21, 22]. We re-constructed the cancer type-specific co-expressed genetic interaction networks for 14 cancer types: bladder urothelial carcinoma (BLCA), breast invasive carcinoma (BRCA), colon adenocarcinoma (COAD), glioblastoma multiforme (GBM), head and neck squamous cell carcinoma (HNSC), kidney renal clear cell carcinoma (KIRC), acute myeloid leukemia (LAML), lung adenocarcinoma (LUAD), lung squamous cell carcinoma (LUSC), ovarian serous cystadenocarcinoma (OV), prostate adenocarcinoma (PRAD), skin cutaneous melanoma (SKCM), thyroid carcinoma (THCA), and uterine corpus endometrial carcinoma (UCEC). Each network contains ~10,000 gene-gene interactions connecting ~4,000 human genes (**S3 Table**). In addition, we collected 2,580,579 nonsynonymous somatic mutations identified from 6,789 tumor exomes across 14 cancer types from TCGA (**S1 Table**), and the tumor distribution in each cancer type was illustrated in **Fig 1B**.

### Network-based Co-mutation measure is a good proxy of tumorigenesis

To evaluate performance of the INCM measure, we next turned to inspect whether genes involved in INCM-predicted putative genetic interactions are highly associated with tumorigenesis. Specifically, we performed a gene-centered enrichment analysis by quantifying the genes’ cumulative co-mutation score (*C*-score) for five known functional gene sets. We calculated the gene’s cumulative *C*-score using cum*C*(*i*) = ∑_*j*∈*N*,*j*≠*i*_*C*(*i*,*j*), where *N* is the total gene sets in the genetic networks and *C*(*i*,*j*) is the *C*-score between gene *i* and *j*, and *C*(*i*,*j*) = 0 if there is no tumor sample with both mutated gene *i* and *j* (*j* belongs to the gene set in the corresponding genetic networks except gene *i*). In the definition of the *C*-score, we posited that genes with high *cum*C value are more likely to be co-mutated with nearby genes in the genetic network in individual tumors (**Fig 1C**). We hypothesized that the high occurrence of the co-mutated gene pairs (termed putative genetic interactions) was correlated with tumorigenesis, and cancer genes would have relatively higher *cum*C value (**Fig 1D**).

**Fig 2:**
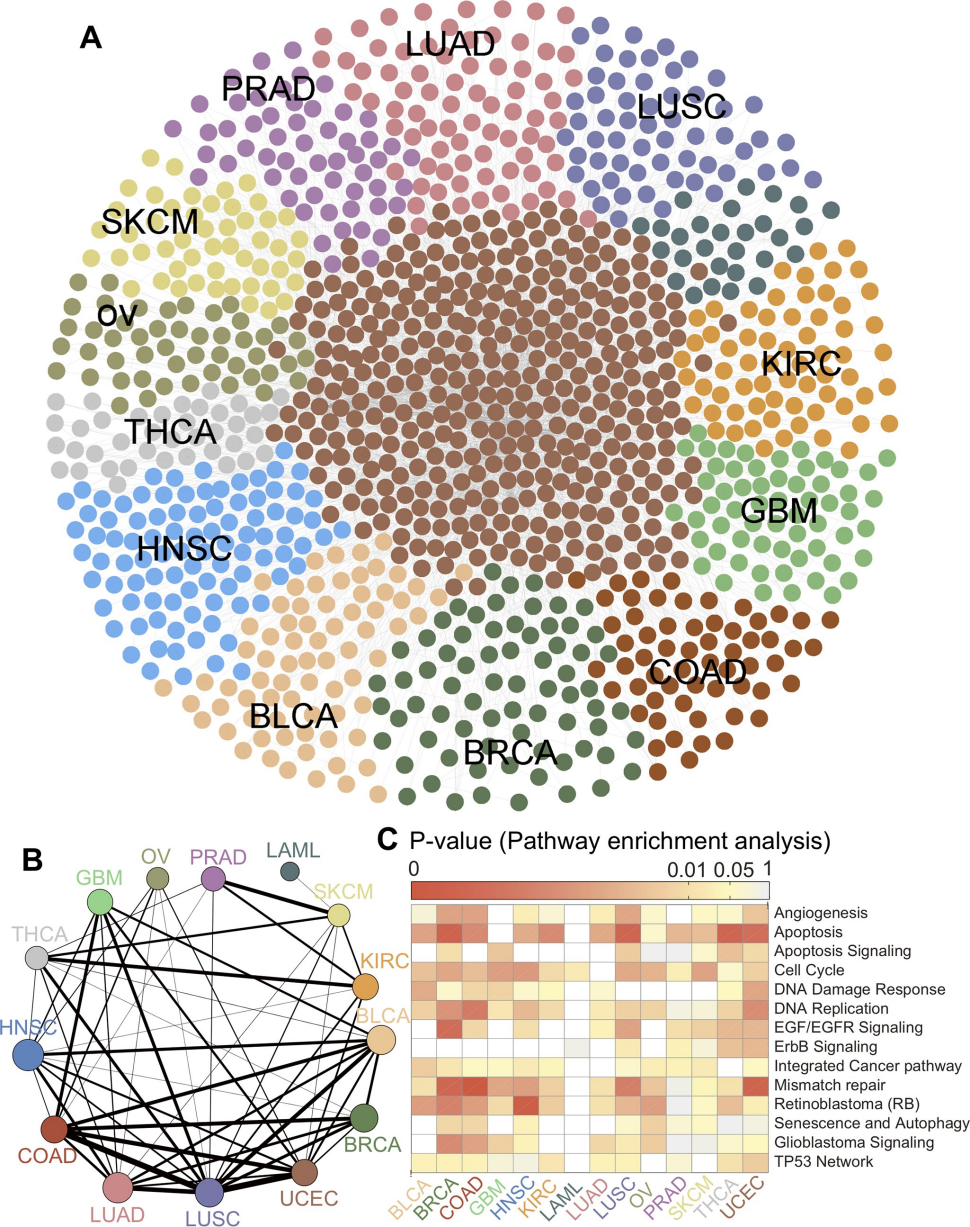
A pan-cancer genetic interaction network. (**A**) Network visualization of a pan-cancer genetic interaction network identified by Individualized Network-based Co-Mutation (INCM) measure across 14 cancer types (**S4 Table**). (**B**) Network overlapping analysis of cancer type-specific genetic interaction networks across 14 cancer types. The node size in *B* represents the number of genes in each cancer type-specific genetic interaction network, and the thickness of the edges between the vertexes represents the number of overlapping genes. (**C**) Canonical cancer pathway enrichment analysis for the INCM-identified cancer type-specific genetic interaction networks across 14 cancer types: bladder urothelial carcinoma (BLCA), breast invasive carcinoma (BRCA), colon adenocarcinoma (COAD), glioblastoma multiforme (GBM), head and neck squamous cell carcinoma (HNSC), kidney renal clear cell carcinoma (KIRC), acute myeloid leukemia (LAML), lung adenocarcinoma (LUAD), lung squamous cell carcinoma (LUSC), ovarian serous cystadenocarcinoma (OV), prostate adenocarcinoma (PRAD), skin cutaneous melanoma (SKCM), thyroid carcinoma (THCA), and uterine corpus endometrial carcinoma (UCEC).

For each gene pair, we compared the network-based correlation for the real mutation patterns and the expected *C*-score (*C*_rand_) when the mutated genes for any individual tumor are reshuffled (**Fig 1D**), where the reshuffle process preserved the mutation frequency of each gene (see Methods). Significant gene pairs (adjusted P–value [q] < 0.05) identified by INCM were selected as putative genetic interactions in cancer and used to further build the final networks (**S4 Table**). **Fig 2A** shows the final pan-cancer genetic network by integrating all 14 INCM-predicted subnetworks in each cancer type. The vertexes in various colors represents the unique genes of the subnetwork in the corresponding cancer type, and the center area represents the overlaps. It should be noted that there is no unique gene in the subnetwork of UCEC. The node size in **Fig 2B** represents the number of genes in each module, and the thickness of the edges between the vertexes represents the number of overlaps. The subnetworks of LUAD, LUSC, COAD and UCEC show significant overlaps, while the subnetworks of OV and LAML seem a little isolated. Functional enrichment analysis shows that genes in the identified genetic subnetwork are significantly enriched in multiple well-known cancer pathways (**Fig 2C**). For example, genes in the INCM-predicted genetic subnetworks are significantly enriched in mismatch repair pathway for BRCA, COAD, LUSC, and UCEC, consistent with a recent pan-cancer study [4]. Put together, the pan-cancer genetic subnetwork identified by INCM offers potential testable hypotheses for understanding tumorigenesis in the context of genetic interactions.

**Fig 3:**
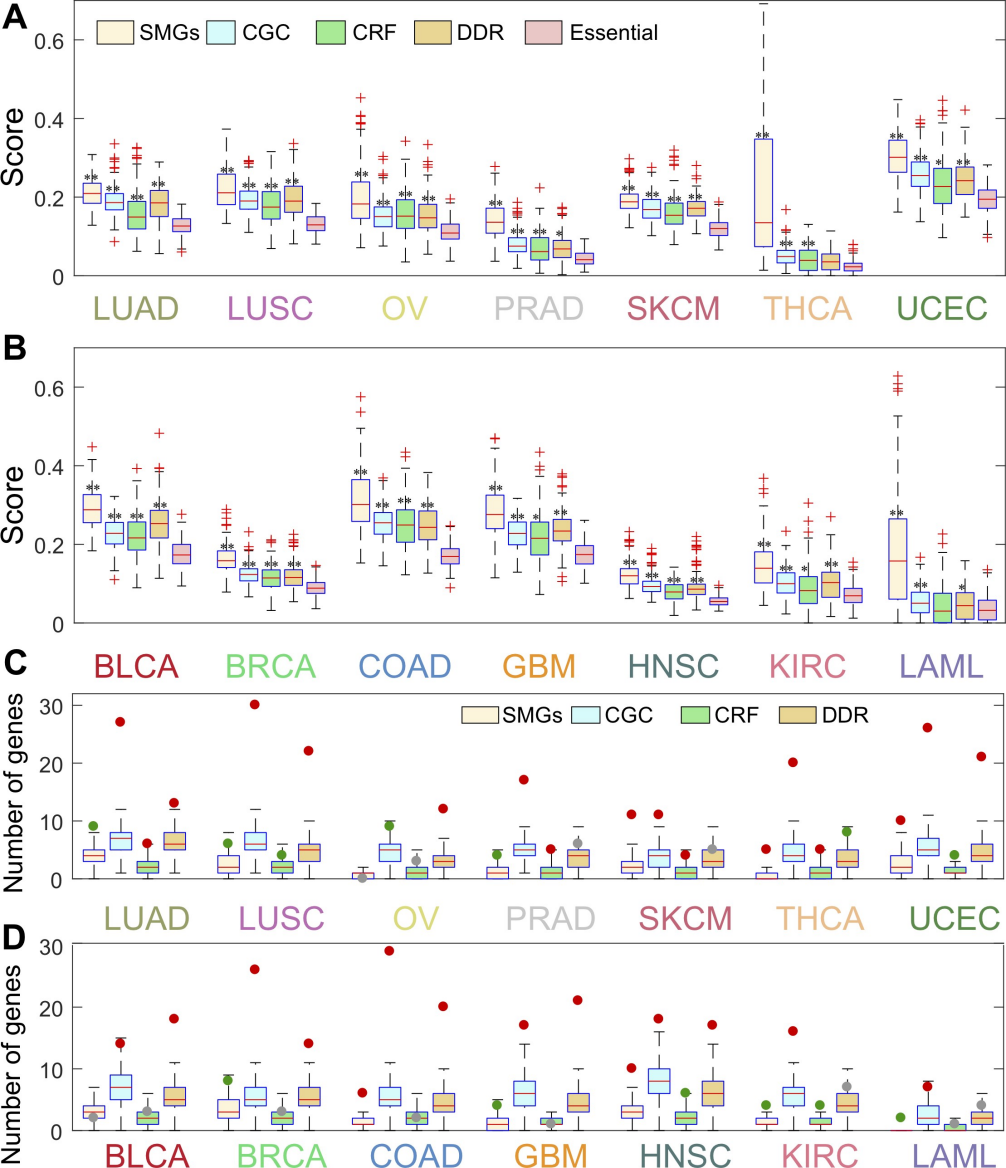
Individualized Network-based Co-Mutation (INCM) measure is a good proxy for tumorigenesis. (**A** and **B**) Boxplots show distribution of INCM-based co-mutation scores for five functional gene sets: significantly mutated genes (SMGs), experimentally validated cancer genes (Cancer Gene Census [CGC]), chromatin regulation factors (CRFs), DNA Damage Repair (DDR) genes, and pan-cancer essential genes across 14 cancer types. P-value was computed by Wilcoxon test. * 0.01<p-value<0.05, ** p-value<0.01. (**C** and **D**) The gene enrichment analysis for genes in the INCM-identified putative genetic interactions across four functional gene sets across 14 cancer types. The box-plots in **C** and **D** are the distribution of the random expectation while the cycles represent the number of the genes for the overlap of the final subnetworks and the cancer related gene sets (red circle: p-value<0.01, green cycle: 0.01<p-value<0.05, and gray cycle: p-value > 0.05). A detailed comparison to random expectation and statistical testing is provided in **S2–S5 Figs**. The full name of 14 cancer types are provided in Fig 2’s legend.

We next turned to inspect the enrichment analysis of genes in the INCM-predicted subnetworks across 14 cancer types using five functional gene sets (**S5 Table**): (i) significantly mutated genes (SMGs) identified in cancer populations collected from over 20 cancer genome analysis projects; (ii) gold-standard experimentally validated cancer (CGC) genes; (iii) DNA Damage Repair (DDR) genes; (iv) chromatin regulation factors (CRFs), and (v) pan-cancer essential genes identified by CRISPR-Cas9 screenings in 324 cancer cell lines across 30 cancer types (see Methods). We found that pan-cancer essential genes (553 genes, **S5 Table**) have a higher *cum*C in comparison to non-essential genes across all 14 cancer types (**S1 Fig**). **Fig 3A** and **3B** illustrate the distribution of *cum*C across five different functional gene sets. Interestingly, we found that the *cum*C of SMGs and CGC were significantly stronger than that of pan-cancer essential genes across all 14 cancer types (*P* < 0.01, two-side Wilcoxon rank-sum test, **S6 Table**). The *cum*C of cancer CRF and DDR genes are significantly stronger than that of pan-cancer essential genes in 13 cancer types except for the LAML. These observations revealed that genes identified in the network-predicted putative genetic interactions by INCM were significantly enriched in cancer-associated gene sets.

We also examined whether the cancer-associated genes were more enriched in the INCM-predicted genetic subnetworks than random gene sets. **Fig 3C** and **3D** illustrated the number of SMGs, CGC genes, CRF genes and DDR genes in the final subnetwork respectively across 14 cancer types. A detailed comparison to random expectation and statistical testing is provided in **S2–S5 Figs**. We found that genes in the INCM-predicted putative genetic interactions were enriched to be in the CGC gene set (*P* < 0.01) across all 14 cancer types, in SMGs across 12 cancer types with the exception of BLCA and OV, in the DDR genes across 12 cancer types with the exception of PRAD and SKCM, and in the CRF genes across 8 cancer types with the exception of BLCA, BRCA, COAD, GBM, LAML and OV (**Fig 3C** and **3D**). Collectively, genes in the putative genetic interactions identified by INCM are enriched significantly in known cancer genes.

### Network-Predicted genetic interactions correlate with patient survival

**Fig 4:**
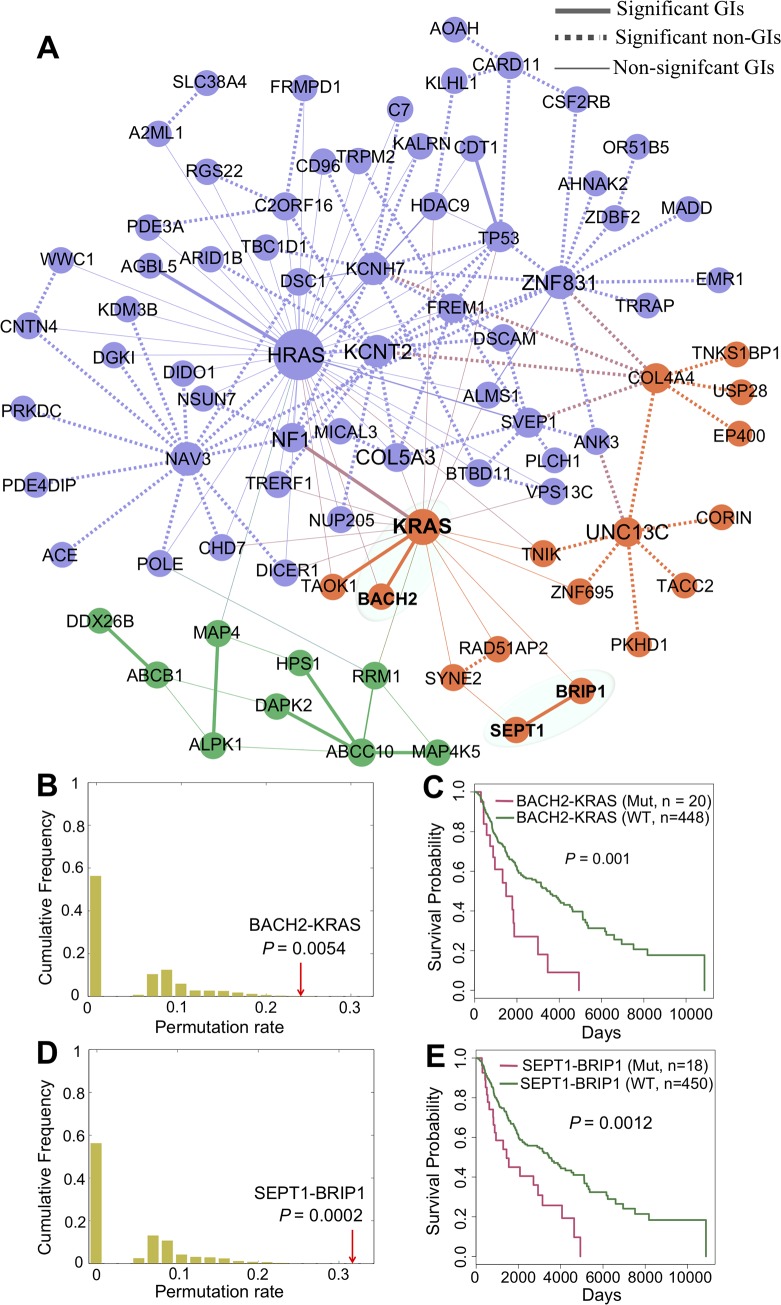
Network-predicted genetic interactions correlate with patient survival in human skin cutaneous melanoma (SKCM). (**A**) The putative genetic interaction network in SKCM identified by individualized network-based co-mutation (INCM) measure. (**B-E**) The INCM-predicted significantly putative genetic interactions correlate with patient survival rate. BACH2-KRAS (**B**) and SEPT1-BRIP1 (**D**) are significantly co-mutated in individual SKCM patients (**S4 Table**). Patients have non-synonymous somatic mutations (Mutant [Mut] group) on genes of BACH2-KRAS (**C**) and SEPT1-BRIP1 (**E**) are significantly correlate with poor survival rate comparing to wild-type [WT] status on both genes. Survival analysis is performed by the “survival” package in R (v3.4.3) and p-value is compared by log-rank test.

We next turned to inspect the correlation of patient survival with network-predicted putative genetic interactions identified by the INCM measure. Here, we focused on SKCM [23] and BRCA [24] as those two cancer types have the high-quality clinical data from TCGA and the sufficient number of network-predicted significant genetic interactions. For SKCM, in total we identified 94 significantly mutated genetic interactions with adjusted q < 0.05 (**S4 Table**). **Fig 4A** illustrates the genetic interaction subnetwork that contains 82 significantly mutated network-predicted genetic interactions for SKCM and 78 known literature-derived genetic interactions connecting 84 genes (**S4 Table**). *KRAS*, encoding the human cellular homolog of a transforming gene isolated from the Kirsten rat sarcoma virus, plays essential roles in multiple cancer types, including melanoma [25, 26]. Here, we identified that *KRAS* showed multiple significant genetic interactions with *BACH2* (q = 0.005, Fig 4B), *TAOK1* (q = 0.006), and *NF1* (q = 0.04). *BACH2*, encoding a 92-kDa transcription factor, belongs to the basic leucine zipper family and control immune function. A recent study reported that BACH2 promoted immunosuppression in cancer. **Fig 4C** reveals that melanoma patients harboring nonsynonymous somatic mutations on *BACH2-KRAS* have poor survival rate compared with the wild-type group (*P* = 0.001, log-rank test). Thus, *BACH2-KRAS* offers a potential target for treatment of cancer by reversing immunosuppression. In addition to *HRAS* and *KRAS*, we also computationally identified several significantly mutated genetic interactions for new gene families, such as *SEPT1-BRIP1* in melanoma (**Fig 4D**). Moreover, patients with nonsynonymous somatic mutations in *SEPT1-BRIP1* reveal poor overall survival compared to the wild type group without somatic mutations on either *SEPT1* and *BRIP1* in SKCM (*P* = 0.0012, log-rank test, **Fig 4E**), offering a potential genetic interaction in melanoma.

For BRCA, in total we identified 82 significantly mutated genetic interactions with adjusted P-value < 0.05 (**S4 Table**). As shown in **S6A Fig**, the putative genetic interaction subnetwork for BRCA contains 19 significantly mutated network-predicted genetic interactions and 259 known literature-derived genetic interactions connecting 135 genes (**S4 Table**). *HRAS*, encoding GTPase HRAS (transforming protein p21), plays important oncogenic roles in multiple cancer types, including breast cancer [25, 27]. However, there are no U.S. FDA-approved drugs for treatment of cancer by direct targeting of HRAS owing to its undruggability [6]. Here, we identified several statistically significant genetic interactions (e.g., *BCL2L1* [*P* = 5.65x10^-3^], **S6B Fig**) for *HRAS* in breast cancer. Interestingly, breast cancer patients harboring nonsynonymous somatic mutations on *BCL2L1-HRAS* reveals poor survival rate compared to the wild-type status on both *BCL2L1* and *HRAS* (*P* = 0, log-rank test, **S6C Fig**). *BCL2L1*, encoding two specific isoforms (Bcl-xL or Bcl-xS), has been shown to have functional roles in multiple cancer types, including breast cancer [28, 29]. APG-1252, a novel Bcl-2/Bcl-XL inhibitor, was considered for the treatment of cancers including small cell lung cancer (SCLC) as investigational new drug by the FDA (NCT03387332). Thus, targeting *BCL2L1-HRAS* by APG-1252 may offer potential therapeutic strategies for treatment of KRAS mutant breast cancer. However, BCL-XL and BCL-XS have different roles in cancer, being aitiapoptotic or proapoptotic, respectively [30]. Further experimental observations are needed to confirm antiapoptotic or proapoptotic roles by targeting BCL2L1-HRAS in human cancer. *XRCC1* (encoding X-ray repair cross-complementing protein 1), a key component of DNA base excision repair, single strand break repair, and backup non-homologous end-joining pathway, was reported to be involved in breast cancer [31, 32]. Herein, *XRCC1* was predicted to have a significant genetic interaction with *HRAS* by network-based INCM co-mutation analysis of individual breast tumors (*P* = 8.45x10^-3^, **S6D Fig**). In addition, our analysis of data related to breast cancer patients harboring nonsynonymous somatic mutations in either gene of XRCC1-HRAS indicates poor survival rate compared with wild-type group (P = 0, log-rank test, **S6E Fig**). *XRCC1* deficiency was reported to promote genomic instability and increase breast cancer risk. We therefore concluded that *XRCC1* deficiency could be exploited for a potential synthetic lethality for *HRAS* in breast cancer [27, 31]. Further studies are needed to provide experimental and clinical validation to decipher the casual relationship of these INCM-predicted putative genetic interactions in cancer.

### Network-Predicted genetic interactions correlate with drug responses

**Fig 5:**
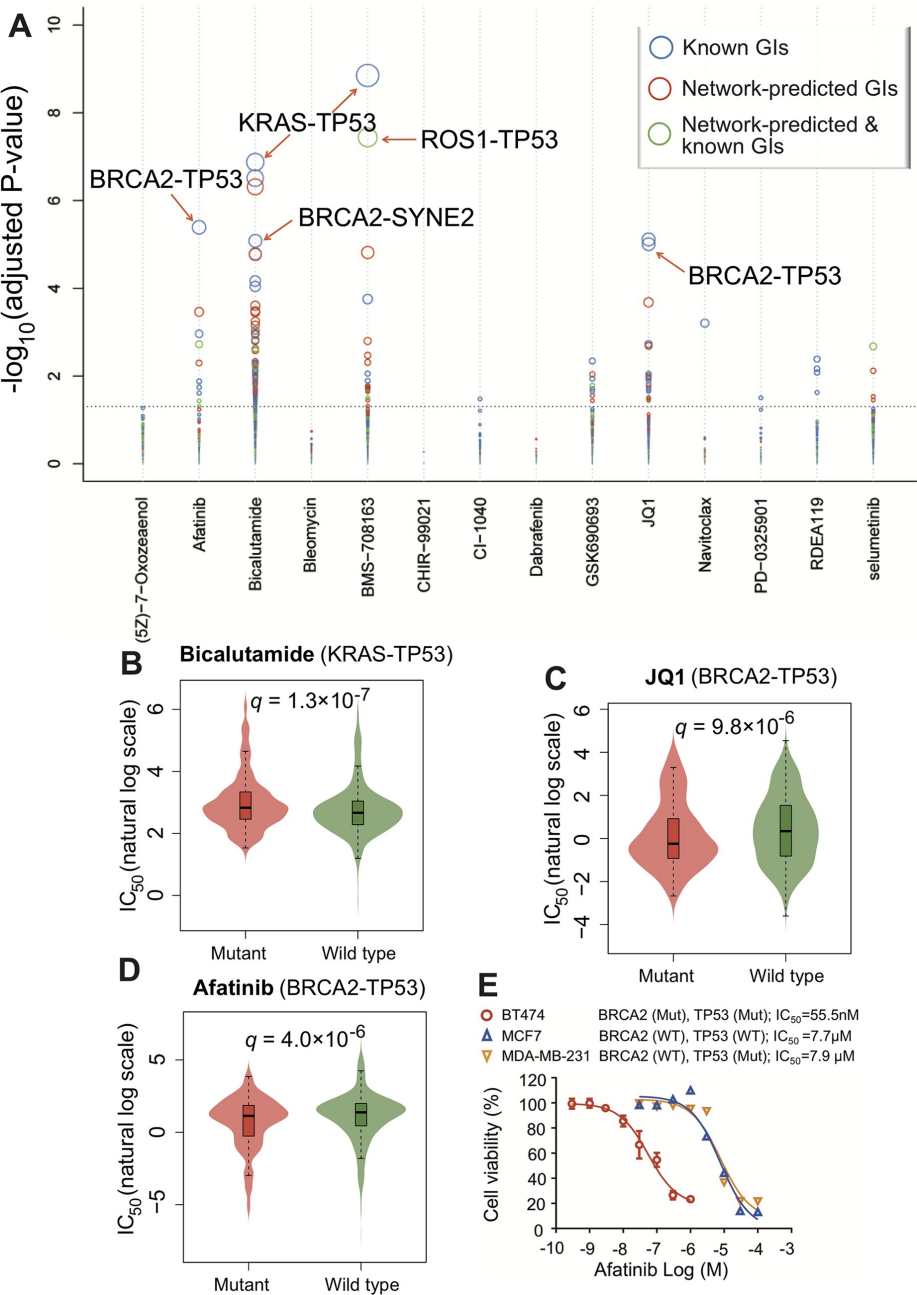
Network-predicted genetic interactions offer cancer pharmacogenomics. (**A**) A bubble plot illustrates that the predicted putative genetic interactions by individualized network-based co-mutation (INCM) measure correlate with drug responses (sensitivity/resistance) for 14 selected anticancer agents. Each dot represents the genetic interaction (gene pair). The size of dots denotes the adjusted P-value (*q*) of relationship between INCM-predicted putative genetic interactions and drug responses. Three types of genetic interactions are illustrated: known genetic interactions (GI), INCM-predicted GI only in individual tumors (Network-predicted GIs), and known GIs and INCM-predicted GI in individual tumors. (**B-D**) Bean plots illustrating two selected genetic interactions (highlighted by arrow in a) correlating with drug resistance or sensitivity of known anti-cancer agents: Bicalutamide (**B**), JQ1 (**C**), and (**D**) Afatinib. The detailed data for **Fig 5A** is provided in the **S7 Table**. P-value is computed using R anova function package (v3.4.3). (**E**) Cell viability assay of afatinib on three breast cancer cell lines with different genotypes: BT474 *(BRCA2* mutant [p.S3094*] and *TP53* mutant [p.E285K]), MCF7 (both *BRCA2* and *TP53* wild-type), and MDA-MB-231 (*BRCA2* wild type but *TP53* mutant [p.R280K]). The half maximal inhibitory concentration (IC_50_) value is determined from the results of at least three independent tests (see Methods).

We next turned to examine the correlation between the network-predicted putative genetic interactions and anticancer drug responses. For each genetic interaction, we used ANOVA to determine if there is significant difference between the cell lines of mutated groups and wild-type groups in terms of their sensitivity/resistance (IC_50_) to the drug. By analyzing drug pharmacogenomic profiles across over 1,000 cancer cell lines from the Genomics of Drug Sensitivity in Cancer (GDSC) database (see Methods), we found that the network-predicted putative genetic interactions were highly correlated to sensitivity/resistance of multiple therapeutic agents (**S7 Table**). **Fig 5A** shows that the network-predicted putative genetic interactions are highly corrected with the sensitivity/resistance of 14 clinically investigational or approved anticancer agents. For example, we found that *KRAS-TP53* were significantly co-mutated in individual tumors across multiple cancer types, such as colon cancer (P < 1.0x10^-4^) and prostate cancer (P = 0.0248, **S4 Table**). Bicalutamide, is a FDA-approved antiandrogen medication for treating advanced prostate cancer [33]. By analyzing cancer pharmacogenomic profiles (see Methods), we found that *KRAS-TP53* are significantly associated with resistance of bicalutamide in cancer cell lines (**Fig 5B**). Previous gene expression analysis revealed that bicalutamide treatment activated the p53 pathway in prostate carcinoma cell lines [34]. In addition, KRAS and androgen receptor synergistically simulate tumor-propagating cells in prostate cancer [35]. Put together, detection of somatic alterations in *KRAS-TP53* may offer a potential biomarker for guiding bicalutamide treatment in prostate cancer.

*BRCA2*, encoding the breast cancer type 2 susceptibility protein, is responsible for repairing DNA in multiple types of cancer cells, including breast cancer [36]. Recent studies reported that *BRCA2* often co-mutates with *TP53* in various cancer types, such as ovarian cancer [37] and breast cancer [38]. We found that the mutation burden is significantly increased in *BRCA2-TP53* co-mutant tumors compared to single-mutant (*BRCA2* or *TP53*) tumors in BRCA (**S7 Fig**). **Fig 5A** and **5D** reveal that cancer cell lines have both somatic mutations on *BRCA2* and *TP53* (*BRCA2-TP53*) are sensitive to afatinib [39]. Afatinib, a dual EGFR and HER2 irreversible inhibitor [39], is under phase II trials in patients with HER2-positive metastatic breast cancer progressing after trastuzumab [40]. To experimentally inspect the sensitivity of afatinib on co-mutant *BRCA2* and *TP53* (*BRCA2-TP53*) breast cancer, we tested cell viability of afatinib on three breast cancer cell lines with different genotypes: BT474 (*BRCA2* mutant [p.S3094*] and *TP53* mutant [p.E285K]), MCF7 (both *BRCA2* and *TP53* wild type), and MDA-MB-231 (*BRCA2* wild-type but *TP53* mutant [p.R280K]). Cell viability assay (see Methods) reveals that afatinib has the strongest cytotoxicity on BT474 (IC_50_ = 55.5 nM, *BRCA2* and *TP53* co-mutant cell line, **Fig 5E**) compared to MCF7 (IC_50_ = 7.7 μM, both *BRCA2* and *TP53* wild-type cell line, **Fig 5E**) and MDA-MB-231 (IC_50_ = 7.9 μM, *TP53* single-mutant cell line, **Fig 5E**), consistent with drug response analysis from the GDSC database (**Fig 5D**).

In addition, co-mutations on *BRCA2* and *TP53* (*BRCA2-TP53*) are sensitive to JQ1 (a BET inhibitor [41]) compared to wild-type cell lines (**Fig 5C**). Clinical studies showed that mutations on *TP53* reduce responsiveness to first-line tyrosine kinase inhibitors in EGFR-mutated non–small cell lung cancer (NSCLC) patients [42]. Pre-clinical studies reported that BET bromodomain inhibition synergized with a PARP inhibitor in BRCA-proficient ovarian cancer cells [43]. In addition to well-known genetic interactions, such as *KRAS-TP53* and *BRCA2-TP53*, several INCM-predicted genetic interactions, including *ROS1-TP53* (**Fig 5A**), *BRCA2-SYNE2* (**Fig 5A**), and *KCNT2-TP53* (**S7 Table**), are significantly associated with responses to multiple anticancer agents. Collectively, INCM-predicted putative genetic interactions offer potential pharmacogenomics biomarkers for guiding personalized cancer treatment.

### Clinically actionable genes are enriched in the network-predicted putative genetic interactions

**Fig 6:**
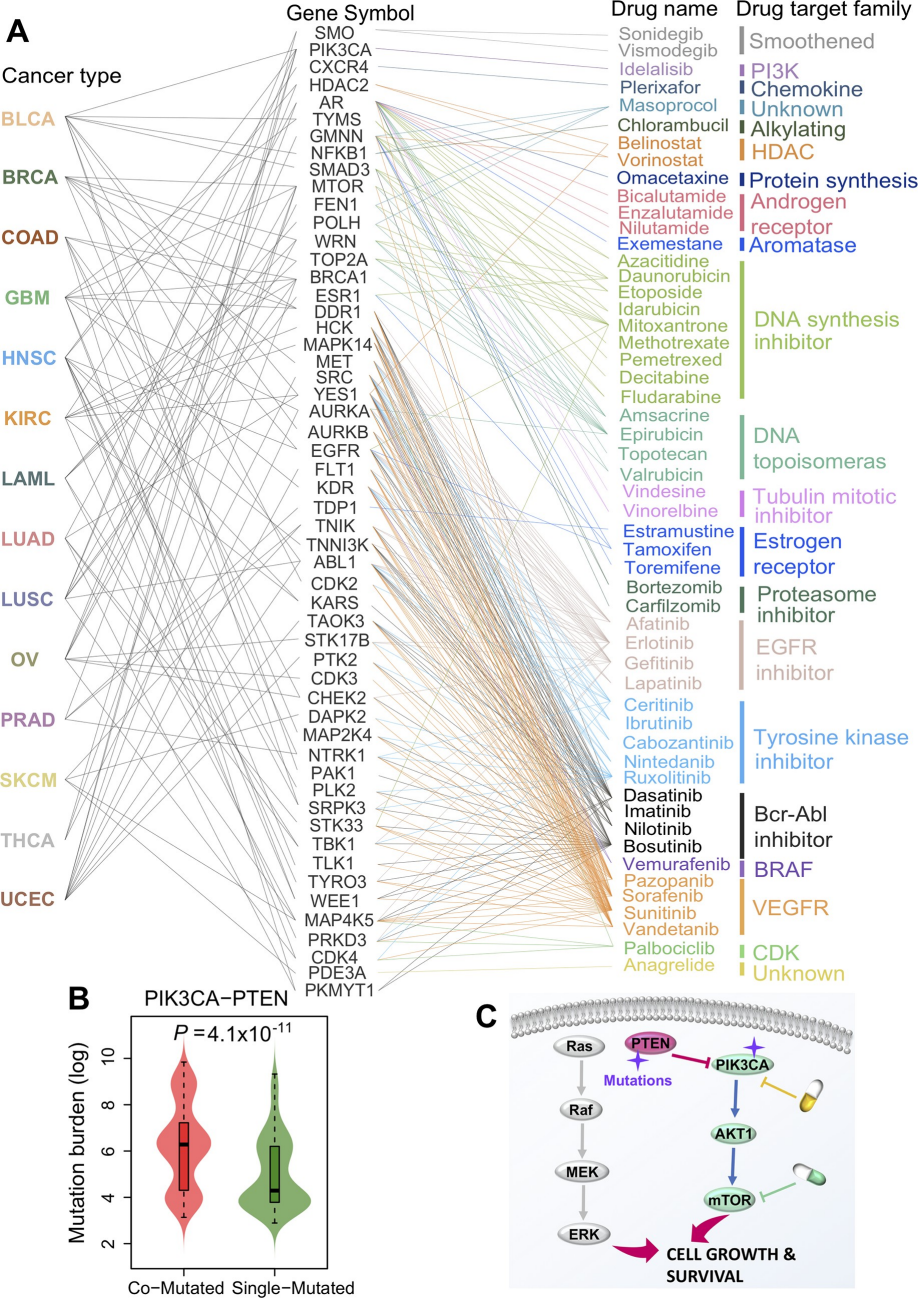
Clinically actionable genes on the network-predicted genetic interactions. (**A**) The mapping between FDA-approved or clinically investigational drugs and their related clinically actionable genes on the putative genetic interactions identified by individualized network-based co-mutation (INCM) measure. The drugs are grouped based on their primary target families (**S8 Table**). (**B**) Mutation burden for PIK3CA-PTEN co-mutant (co-mutated) tumors compared to single-mutant tumors on *PIK3CA* or *PTEN* alone in uterine corpus endometrial carcinoma (UCEC). P-value was computed by Wilcoxon test. (**C**) A proposed pharmacogenomics model for drug sensitivity/resistance mechanism of somatic mutations on *PTEN-PIK3CA* in human cancers. *PIK3CA-PTEN* (P < 1.0x10^-4^) was significantly co-mutated in UCEC identified by INCM measure. The full name of 14 cancer types are provided in Fig 2’s legend.

We further inspected the clinical implications of the network-based predicted putative genetic interactions by performing drug-target network analysis. Here we focused on 94 FDA-approved or chemotherapeutic or molecularly targeted agents (see Methods). The targets of drugs were grouped into 21 families as shown in **Fig 6A**. We found that genes in the INCM-predicted putative genetic interactions can be targeted by FDA-approved drugs across multiple therapeutic pathways (**S8 Table**), such as PI3K inhibitors (idelalisib), HDAC inhibitors (vorinostat and belinostat), proteasome inhibitors (bortezomib, carfilzomib, and lxazomib), tyrosine kinase inhibitors (i.e., ceritinib and ruxolitinib), and CDK inhibitor (palbociclib). Palbociclib is the first FDA-approved CDK4/6 inhibitor for the treatment of estrogen-positive and HER2-negative breast cancer [44]. Here we found that *CETN2-CDK4* (P = 0.018) are significantly co-mutated in individual ovarian tumors. *CETN2*, an X-linked gene encoding centrin, plays important roles in the centrosome of human cells. Down-regulation of CETN2 was reported to be involved in tumor suppressive functions in bladder cancer [45]. In addition, phosphorylation of centrin during the cell cycle process preceded centrosome duplication [46], and centrosome duplication played essential roles in genomic instability and cancer [47]. Thus, network-based INCM analysis generates a hypothesis for a potential synthetic lethal interaction for CETN2 and CDK4 co-mutated ovarian cancer. Idelalisib, is an FDA-approved PIK3CA inhibitor for the treatment of patients with follicular lymphoma and small lymphocytic lymphoma [48]. Here, we found that 159 samples have somatic mutations in both *PTEN* and *PIK3CA* (*PIK3CA-PTEN* [P < 1.0x10^-4^] by INCM analysis) in UCEC from TCGA. In addition, the mutation burden is significantly increased in *PIK3CA-PTEN* co-mutated samples compared to tumors with single-mutant *PTEN* or *PIK3CA* alone in UCEC (**Fig 6B**). Recently early-phase trials showed that PI3K/AKT/mTOR inhibitors appear promising for the treatment of *PIK3CA* and *PTEN* altered tumors based on 1,656 patients from MD Anderson cancer center [49]. Thus, detection of co-mutations of both *PIK3CA* and *PTEN* may offer potential biomarkers or targets for individualized treatment of tumors in uterine cancer or other cancer types (**Fig 6C**). Further clinical studies will be necessary to conduct before clinical applications. In summary, network-based predicted putative genetic interactions identified here offer potential therapeutic targets (**Fig 6**) for development of personalized cancer medicine.

## Discussion

Here we proposed a novel individual co-mutation analysis approach, named INCM, to infer putative genetic interactions to uncover novel therapeutic targets or actionable pharmacogenomic biomarkers. Integrating large-scale somatic mutations with known genetic interaction networks, INCM can be used to build cancer type-specific genetic subnetworks which are significantly enriched in known cancer genes and well-established cancer pathways. In addition, we showed that several selected putative genetic interactions identified by INCM were significantly associated with patient survival and drug responses, offering potential biomarkers for guiding tumor diagnosis and cancer pharmacogenomics studies.

Despite many strengths, our approach has several potential pitfalls. INCM is focused on the co-mutation analysis principle across individual tumors; thus, it is powerful for cancer types with high mutation burden, like colon and uterine cancers. That however is not the case for cancer types with overall low mutation burden, such as LAML or THCA (**Fig 3**). Second, INCM is built based on the known genetic interactions derived from human or homologous genes from organisms. The network incompleteness and potential literature data bias may lead to potential false positive rates and low robustness in quantifying INCM measure. A large-scale, systematic genetic interactome [50], may provide an unbiased genetic interactome network for our future study. Early-stage and late-stage diseases have different tumor biology, distinct mutational profiles, diverse tumor heterogeneity, drug response, and survival curves. Mixing tumors with distinct molecular subtypes and different driver mutational landscapes are potential weakness. Each INCM-inferred genetic interaction has different tissue-specific presentation, disease penetrance, and distinct tumorigenicity spectrum in different organs and tissue types. Mixing them together in the INCM enrichment analyses without any detailed clinical and pathological descriptions (e.g., tumor grade, size, pathological stage, recurrence, and local and distance metastasis in each specific cancer type), may limit its clinical impact and translational application in the current study. The co-existence of multiple INCM genes is often the result of loss of genome integrity (p53 mutation) and compromised DNA repair capacity. In addition, our INCM analysis tends to focus on the rarely mutated genes for the definition of the network-based correlation. Thus, several well-known major oncogenes with high mutation frequency are missed in current INCM framework. Building the causal relationship of genetic interactions reminds challenge in current INCM framework by the lack of the dedicated functional data. Finally, this study only integrated 2.5 million nonsynonymous somatic mutations (e.g., missense mutations and small indels) from the TCGA project. For example, hematological cancers chromosome rearrangements [51], such as BCR-ABL fusion (the Philadelphia chromosome), cannot be identified in current INCM analysis. The publicly available copy number variation profiles, gene fusions, and large-scale somatic mutations from TCGA pan-cancer project [4, 52] and ICGC project [53] would significantly enhance the applications of INCM in the future. In addition, we used all nonsynonymous somatic mutations (including driver mutations and passenger mutations) in current INCM analysis. Filtering passenger mutations using bioinformatics tools, such as deleterious mutations derived by the combined annotation dependent depletion (CADD) score [54], may improve performance of INCM further. Finally, identification of genetic interactions with context specific lethality will be more important as opposed to genes whose deletion are universally lethal, which may offer highly selective targets for personalized antitumor drug development.

In summary, this study offers a powerful systems biology-based methodology for comprehensive identification of candidate therapeutic pathways that potentially target tumor vulnerabilities and prioritization of potential pharmacogenomics biomarkers for development of personalized cancer medicine. To the best knowledge of the authors, this is the first large-scale, pan-cancer genetic interaction network study that integrates the largest tumor whole-exome sequencing and RNA-seq datasets for identification of candidate therapeutic pathways that target tumor vulnerabilities and for prioritization of potential diagnostic and pharmacogenomics biomarkers. From a translational perspective, if broadly applied, the systems biology-based methodology developed here can minimize the translational gap between genomic studies and clinical outcomes in cancers, which is a significant problem in personalized medicine.

## Materials and methods

### Somatic mutation profiles in primary tumors

We downloaded the somatic mutation data from TCGA GDC Data Portal (https://portal.gdc.cancer.gov/). Variant calls from MuTect2 pipeline were used in this study. We only focused on somatic mutations in TCGA tumor-normal matched samples across 14 cancer types (**S1 Table**).

### Cancer cell line annotation

We downloaded putative somatic mutations for 1,001 cancer cell lines from the Genomics of Drug Sensitivity in Cancer (GDSC, http://www.cancerrxgene.org/). The list of genomic variants found in these cell lines by whole exome sequencing was also obtained from GDSC. Sequencing variants were identified by comparing to a reference genome. The resulting variants were then filtered using the data from NHLBI GO Exome Sequencing Project and the 1000 Genomes project to remove sequencing artefacts and germline variants [55].

### Collection of drug response data

Natural log half maximal inhibitory concentration (IC_50_) and the area under the dose-response curve (AUC) values for all screened cell line/drug combinations were downloaded from GDSC (http://www.cancerrxgene.org/). After applying the data preparation procedure described in a previous study [55], a total of 251 drugs tested in 1,074 cancer cell lines with 224,510 data points were used.

### Statistical analysis of drug responses

For each drug, an ANOVA model was fitted to correlate drug response with the mutation status of each genetic interaction across all cell lines. Linear model was used with IC_50_ values as dependent variables and factors including tissue type, screening medium, and micro-satellite instability status in addition to the mutation status. A genomic feature-drug pair was tested only if the final drug-response vector contained at least 3 positive cell lines and at least 3 negative cell lines. Effect size was quantified through the Cohen’s *d* using the difference between two means divided by a pooled standard deviation for the data. The resulting p-values were corrected (on each drug basis) with Benjamini-Hochberg Procedure [56]. The ANOVA was performed using R anova function package.

### Construction of cancer type-specific co-expressed network

Here, we assembled the known genetic interactions from three data sources [57–59]. In this study, we used the experimentally validated genetic interactions in both human and non-human cells or models and organisms (**S2 Table**). For non-human organisms, we use the human-organism homologous genes from OrthoDB [60]. In total, we collected ~21,000 genetic interactions connecting ~6,600 genes for each cancer type. We next reconstructed the cancer type-specific co-expressed genetic interaction network by mapping RNA-seq data (RPKM) from 14 cancer types. Normalized gene expression data from RNA-Seq (RPKM) were extracted using the R package from TCGA-Assembler [61]. We computed the co-expression (Pearson correlation coefficient) and p-value (F-statistics) for each genetic interaction (gene-gene pair) based on RNA-seq data in each cancer type. Finally, we only used the p-value (P) < 0.05 as co-expressed genetic interactions to build the cancer type-specific co-expressed genetic interaction network for each cancer type.

### Description of network-based mathematical framework

Here we inferred the genetic interaction by searching novel co-mutation patterns of gene pairs across individual tumors from the known genetic network architecture (**Fig 1A**). For a gene *i*, let *G*(*i*) denote the set of tumors with mutated gene *i* for the given cancer type (**Fig 1B**). And for each gene pair, we can calculate the value of the network-based correlation in Eq (1) to illustrate the gene interaction in the corresponding cancer type (**Fig 1C**), where |*G*(*i*)∩*G*(*j*)| represents the number of tumors where both genes *i* and *j* are the mutated gene (the number of the tumors overlap of *G*(*i*) and *G*(*j*)), *m*(*i*) and *m*(*j*) are the cumulative mutations of genes *i* and *j* respectively and *d*(*i*,*j*) is the distance (shortest path length) between genes *i* and *j* in the genetic network for the given cancer type. The measure based on the somatic mutations $\frac{\left|G(i)\cap G(j)\right|}{\sqrt{m(i)\times m(j)}}$, which is also considered as the Salton index, indicates the probability of co-mutation between gene pairs in the same individual tumor, and the adjustment by cumulative mutations can to some extent eliminate the potential bias from frequently mutated genes. From this definition, two genes, which are more likely to be the mutated genes in the same tumor and close to each other in the genetic network, generate a large *C*-score.

In order to assess the significance of the *C*-score between two genes, we create a reference *C*-score distribution corresponding to the *C*-score for the simulation with shuffling the mutated genes for each individual tumor. For a given cancer type, we can infer the mutation probability as $p(i)=\frac{m(i)}{\sum_{i\in N}m(i)}$ for gene *i*. For an individual tumor *t* for a given cancer type in our dataset, we denote *l*(*t*) as the number of the mutated genes. We reshuffle its mutated genes by randomly generating a mutated gene list with *l*(*t*) non-overlapping genes. The selection of gene *i* depends on its mutation probability *p*(*i*). The process is terminated when all the tumors’ mutated genes are reshuffled. It should be noted that the cumulative mutation times of each gene remain unchanged in this simulation process, but the mutated genes in each individual tumor are totally changed, leading to different co-mutated correlation between gene pairs. The reference *C*-score distribution was generated by calculating the *C*-score based on the reshuffled mutations for each individual tumor, and the process was independently repeated 10,000 times. In this case, we can define the significance (P-value) of each gene pairs using per-mutation calculation: $P_{ij}=\frac{\left|C_{R}>C(i,j)\right|}{10^{4}}$, where |*C*_*R*_>*C*(*i*,*j*)| is the number of times that the simulated *C*-score is larger than the real systems (**Fig 1D**).

We sorted all gene pairs with an ascending order of significance of the *C*-score for the given cancer type. We selected the top 100 gene pairs (except gene pairs whose cumulative mutations equal to 1, which may bias the significance), as well as the interaction between these corresponding genes in the original genetic network. The largest component of these selected genetic interactions is considered as the final module, which can be used as potential diagnosis or pharmacogenomics biomarkers.

### Collection of functional gene sets

Cancer Gene Census (CGC). We collected a large number of cancer genes from several publicly available sources. First, a total of 563 genes (**S5 Table**) were downloaded from Cancer Gene Census [62] (denoted as CGC genes). CGC genes are well-curated and have been widely used as a reference cancer gene set in many cancer-related projects [63].

Significantly Mutated Genes (SMGs). We collected 693 significantly mutated genes (SMGs) in cancer (**S5 Table**) from TCGA publications and several other publications as described in our previous study [64].

DNA Damage Repair (DDR) Genes. DNA damage response processes of relevance from cancer DNA repair pathway, whose processes are crucial for the maintenance of genetic information in the cancer genome [65]. Here we compiled 276 DDR genes (**S5 Table**) from a recent TCGA pan-cancer analysis project [66].

Chromatin Regulation Factors (CRFs). The CRFs that modulate the epigenetic landscape have emerged as potential gatekeepers and signaling coordinators for the maintenance of genome integrity, involved in development of cancer [67]. We downloaded 176 genes encoding CRFs (**S5 Table**) from our previous study [22].

Pan-cancer Essential Genes. We compiled the genome-wide human essential genes from a high-resolution CRISPR-Cas9 screens in 324 human cancer cell lines from 30 cancer types [68]. In total, we used 553 pan-cancer core fitness genes (**S5 Table**) as tumor cell-line essential genes defined in the previous study [68].

### Patient survival analysis

We downloaded patients’ clinical information using the R package from TCGA-Assembler [61]. Survival analysis was conducted using “survival” package in R (v3.4.3) by correlating patient’s overall survival with the somatic mutation status of each genetic interaction. The statistical significance was calculated using the logrank test.

### Cell viability assay

Afatinib was purchased from Medchem express (Monmouth Junction, NJ). Breast cancer cell lines BT474, MCF-7 and MDA-MB-231 were obtained from the American Type Culture Collection. BT474 and MDA-MB-231 cells were cultured in DMEM medium and MCF-7 cells were cultured in RPMI-1640 medium and both mediums were supplemented with 10% fetal bovine serum (FBS), 100 unit/ml penicillin and 100 μg/ml streptomycin (Thermo Fisher Scientific, Waltham, MA). All cells were maintained at 37°C in a humidified incubator containing 5% CO2. Cancer cells were seeded at a density of 3,000–5,000 per well in 96-well plates and then treated with indicated concentrations of afatinib for 72 h. Cell viability was evaluated using CellTiter 96 AQueous One Solution Cell Proliferation kit (Promega, Madison, WI) according to the manufacturer’s protocol. The half maximal inhibitory concentration (IC_50_) value was determined from the results of at least three independent tests.

### Network visualization, statistical analysis, and pathway enrichment analysis

The statistical analysis in this study was carried out on R platform (v3.4.3, http://www.r-project.org/). Networks were visualized by Gephi (v0.9.2, https://gephi.org). Pathway enrichment analysis was performed by ClueGO [69].

## Supporting information

S1 Fig Distribution of *cum*C (C) for the tumor cell-line essential genes identified by CRISPR-Cas9 screenings in cancer cell lines comparing to non-essential genes. The red line is the average value of the *cum*C for the tumor-cell line essential genes (553 genes, **S5 Table**) and the shadow represent the distribution of the average *cum*C of the matching number of non-essential genes by 10,000 times random sampling. The number of genes for each random sample is equal to the essential genes. P-value was computed by permutation test.
(PDF)

S2 Fig The significantly mutated gene (SMGs) enrichment analysis for genes in the Individualized Network-based Co-Mutation (INCM) measure-identified putative genetic interactions across 14 cancer types. Red lines represent the number (n) of the overlapped genes between SMGs (**S5 Table**) and genes in the INCM-identified putative genetic interaction network. Bar graphs represent 10,000 times random sampling and the number of genes in each sampling test is equal to the number of genes in the INCM-identified putative genetic interaction network. P-value was computed by permutation test.
(PDF)

S3 Fig The Cancer Gene Census (CGC) gene enrichment analysis for genes in the Individualized Network-based Co-Mutation (INCM) measure-identified putative genetic interactions across 14 cancer types. Red lines represent the number (n) of the overlapped genes between CGC genes (**S5 Table**) and genes in the INCM-identified putative genetic interaction network. Bar graphs represent 10,000 times random sampling, and the number of genes in each sampling test is equal to the gene set of the INCM-identified putative genetic interaction network. P-value was computed by permutation test.
(PDF)

S4 Fig The chromatin regulation factors (CRFs)-coding gene enrichment analysis for genes in the Individualized Network-based Co-Mutation (INCM) measure-identified putative genetic interactions across 14 cancer types. Red lines represent the number (n) of the overlapped genes between CRFs coding genes (**S5 Table**) and genes in the INCM-identified putative genetic interaction network. Bar graphs represent 10,000 times random sampling, and the number of genes in each sampling test is equal to the gene set of the INCM-identified putative genetic interaction network. P-value was computed by permutation test.
(PDF)

S5 Fig The DNA damage repair gene enrichment analysis for genes in the Individualized Network-based Co-Mutation (INCM) measure-identified putative genetic interactions across 14 cancer types. Red lines represent the overlapped number (n) of genes between DNA damage repair genes (**S5 Table**) and genes in the INCM-identified putative genetic interaction network. Bar graphs represent 10,000 times random sampling, and the number of genes in each sampling test is equal to the gene set of the INCM-identified putative genetic interaction network. P-value was computed by permutation test.
(PDF)

S6 Fig INCM-predicted genetic interactions correlate with patient survivals in breast invasive carcinoma (BRCA). (**A**) The putative genetic interaction network in BRCA identified by individualized network-based co-mutation (INCM) measure. (**B**-**D**) The identified significantly putative genetic interactions correlate with patient survival rate. BCL2L1-HRAS (**B**) and XRCC1-HRAS (**D**) are significantly co-mutated BRCA patients. Patients have mutations (Mutant [Mut] group) on both genes of BCL2L1-HRAS (**C**) or XRCC1-HRAS (**E**) are significantly correlate with poor survival rates comparing to wild-type [WT] group on both genes. P-value in **B** and **D** was computed by permutation set. P-value in **C** and **E** was computed by log-rank test.
(PDF)

S7 Fig Mutation burden for co-mutant (Co-mutated) tumors compared to single-mutant (Single-Mutated) tumors on BRCA2-TP53 and PIK3CA-PTEN in breast invasive carcinoma (BRCA). (PDF)

S1 Table The statistic of somatic mutation profiles across 14 cancer types collected from The Cancer Genome Atlas (TCGA) project. (PDF)

S2 Table The cancer type-specifically co-expressed genetic interactions for 14 cancer types. All genetic interactions were identified by experimental assays in organisms or human cells or models.
(XLSX)

S3 Table The statistics of the cancer type-specifically co-expressed genetic networks for 14 cancer types. (PDF)

S4 Table The final genetic interactions networks for 14 cancer types identified by Individualized Network-based Co-Mutation (INCM) analysis. (XLSX)

S5 Table The detailed lists of five functional gene sets: Significantly mutated genes (SMGs), Cancer Gene Census (CGC), DNA damage repair (DDR) genes, Chromatin regulation factors (CRFs), and human essential genes. (XLSX)

S6 Table Average *cum*C value for five functional gene sets. The P values are calculated by the Wilcoxon rank-sum test over the corresponding gene set and human essential genes identified in cancer cell lines.
(PDF)

S7 Table The predicted association between the network-predicted putative genetic interactions and the drug responses (sensitivity/resistance) for multiple therapeutic agents using ANOVA. (XLSX)

S8 Table The list of therapeutic pathways for the INCM-predicted putative genetic interactions targeted by FDA-approved drugs using drug-target network analysis. (XLSX)
