# Supplementary material for: Individualized genetic network analysis reveals new therapeutic vulnerabilities in 6,700 cancer genomes
Source: PLoS Comput Biol. 2020 Feb 26;16(2):e1007701. doi: 10.1371/journal.pcbi.1007701 (PMC7062285; doi:10.1371/journal.pcbi.1007701)
Supplement: S1 Fig — The red line is the average value of the cumC for the tumor-cell line essential genes (553 genes, S5 Table) and the shadow represent the distribution of the average cumC of the matching number of non-essential genes by 10,000 times random sampling. The number of genes for each random sample is equal to the essential genes. P-value was computed by permutation test. (PDF) [file pcbi.1007701.s001.pdf]

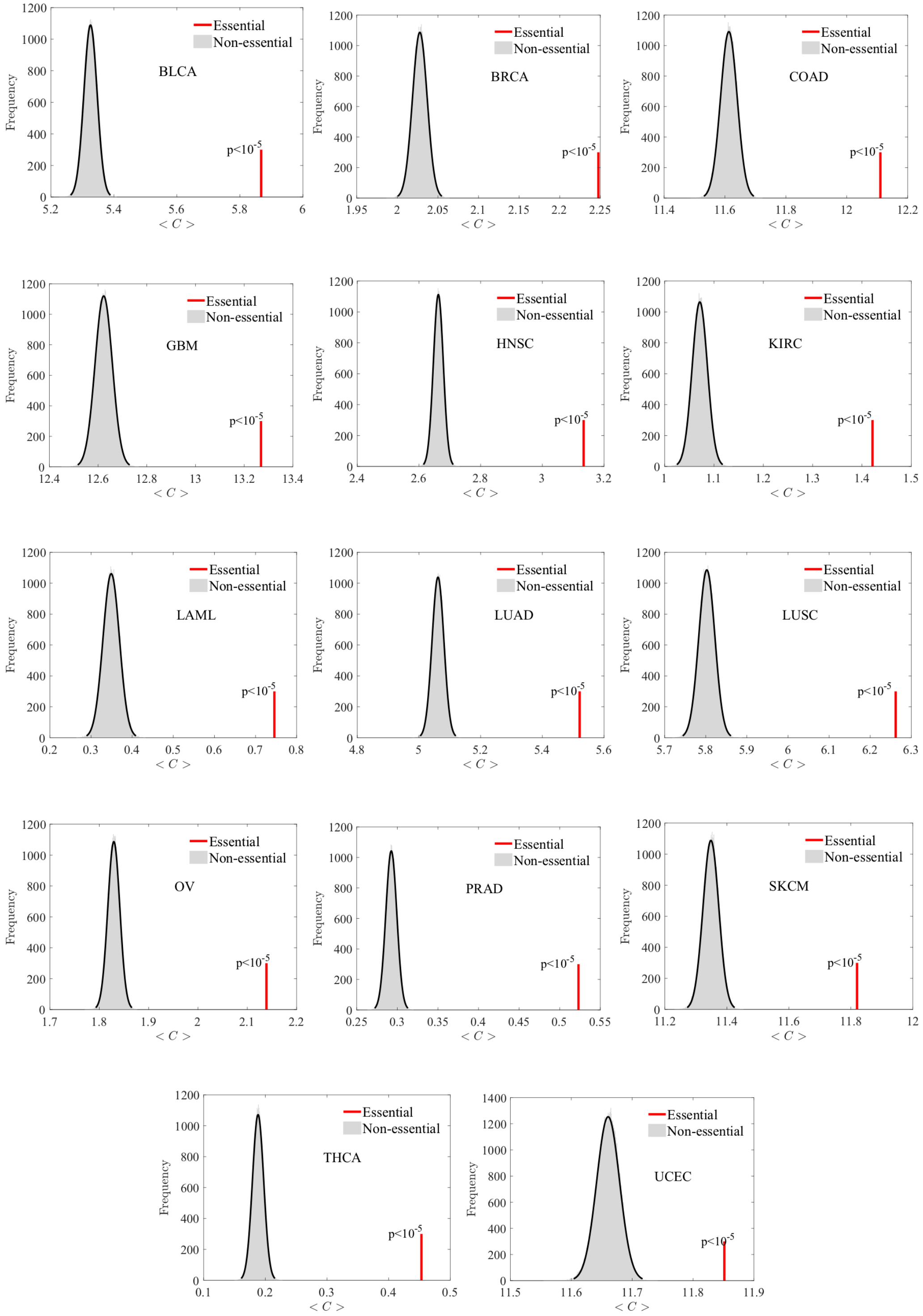

S1 Fig. Distribution of cum*C* (*C*) for the 553 pan-cancer essential genes identified by CRISPR-Cas9 screenings in 324 cancer cell lines compared to non-essential genes. The red line is the average value of the *cumC* for the essential genes and the shadow represent the distribution of the average *cumC* of the non-essential genes by 10,000 times random sampling. The number of genes for each random sample is equal to the pan-cancer essential genes.
