# Supplementary material for: Individualized genetic network analysis reveals new therapeutic vulnerabilities in 6,700 cancer genomes
Source: PLoS Comput Biol. 2020 Feb 26;16(2):e1007701. doi: 10.1371/journal.pcbi.1007701 (PMC7062285; doi:10.1371/journal.pcbi.1007701)
Supplement: S2 Fig — Red lines represent the number (n) of the overlapped genes between SMGs (S5 Table) and genes in the INCM-identified putative genetic interaction network. Bar graphs represent 10,000 times random sampling and the number of genes in each sampling test is equal to the number of genes in the INCM-identified putative genetic interaction network. P-value was computed by permutation test. (PDF) [file pcbi.1007701.s002.pdf]

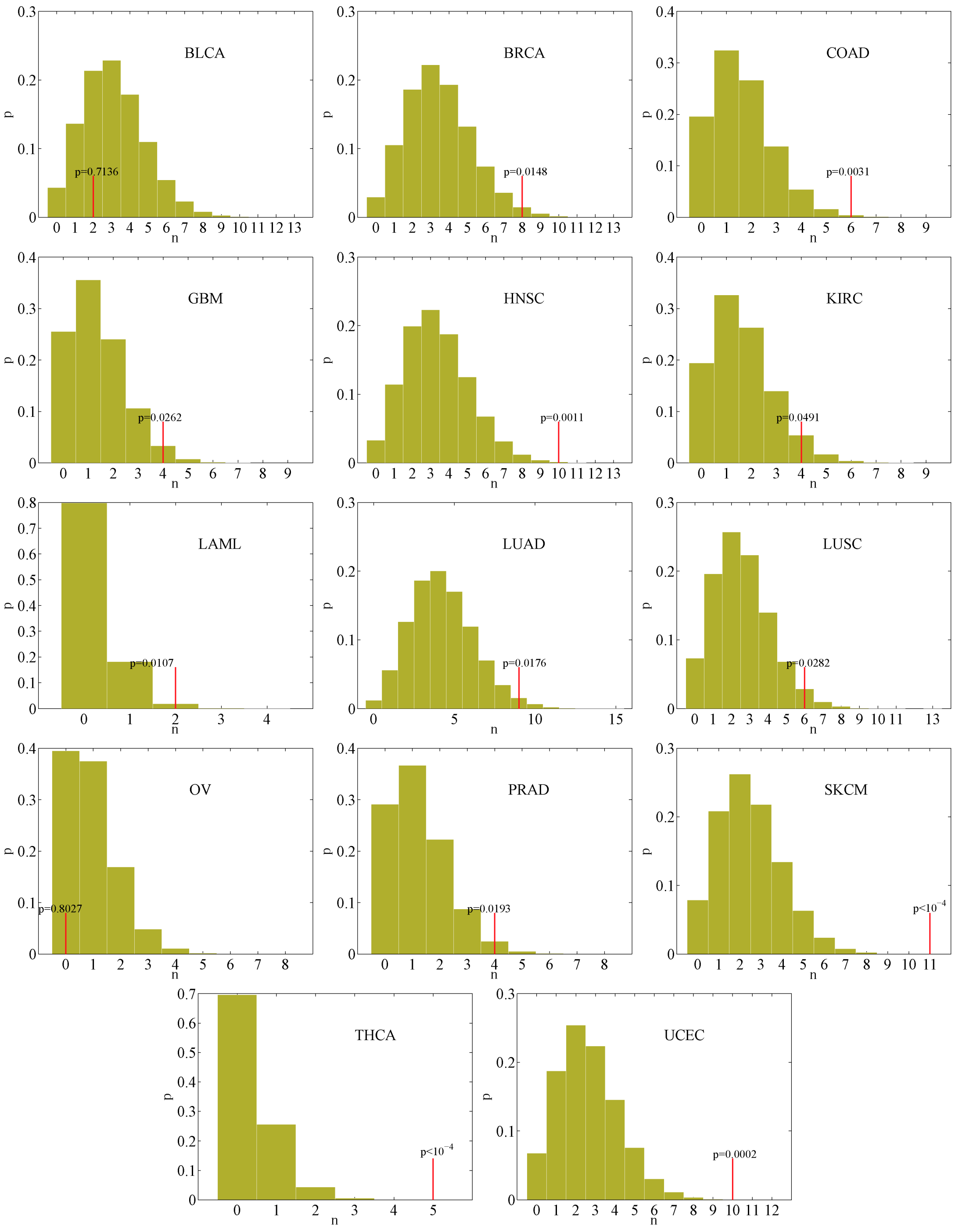

S2 Fig. The significantly mutated gene (SMGs) enrichment analysis for genes in the Individualized Network-based Co-Mutation (INCM) measure-identified putative genetic interactions across 14 cancer types. Red lines represent the number (n) of the overlapped genes between SMGs (**S5 Table**) and genes in the INCM-identified putative genetic interaction network. Bar graphs represent 10,000 times random sampling and the number of genes in each sampling test is equal to the number of genes in the INCM-identified putative genetic interaction network. P-value was calculated by permutation test.
