# Supplementary material for: Individualized genetic network analysis reveals new therapeutic vulnerabilities in 6,700 cancer genomes
Source: PLoS Comput Biol. 2020 Feb 26;16(2):e1007701. doi: 10.1371/journal.pcbi.1007701 (PMC7062285; doi:10.1371/journal.pcbi.1007701)
Supplement: S3 Fig — Red lines represent the number (n) of the overlapped genes between CGC genes (S5 Table) and genes in the INCM-identified putative genetic interaction network. Bar graphs represent 10,000 times random sampling, and the number of genes in each sampling test is equal to the gene set of the INCM-identified putative genetic interaction network. P-value was computed by permutation test. (PDF) [file pcbi.1007701.s003.pdf]

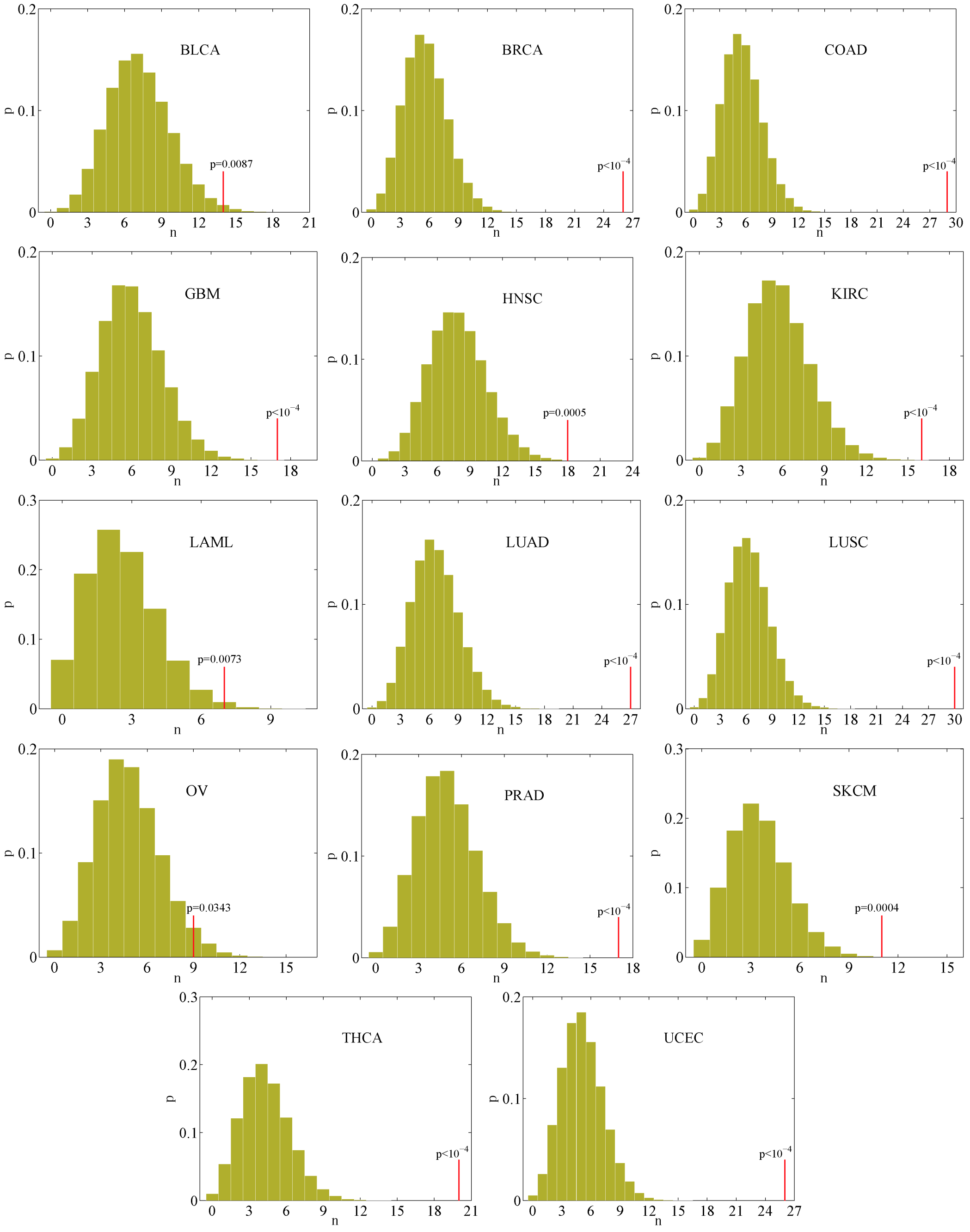

S3 Fig. The Cancer Gene Census (CGC) gene enrichment analysis for genes in the Individualized Network-based Co-Mutation (INCM) measure-identified putative genetic interactions across 14 cancer types. Red lines represent the number (n) of the overlapped genes between CGC genes (**S5 Table**) and genes in the INCM-identified putative genetic interaction network. Bar graphs represent 10,000 times random sampling, and the number of genes in each sampling test is equal to the gene set of the INCM-identified putative genetic interaction network. P-value was calculated by permutation test.
