# Supplementary material for: Individualized genetic network analysis reveals new therapeutic vulnerabilities in 6,700 cancer genomes
Source: PLoS Comput Biol. 2020 Feb 26;16(2):e1007701. doi: 10.1371/journal.pcbi.1007701 (PMC7062285; doi:10.1371/journal.pcbi.1007701)
Supplement: S6 Fig — (A) The putative genetic interaction network in BRCA identified by individualized network-based co-mutation (INCM) measure. (B-D) The identified significantly putative genetic interactions correlate with patient survival rate. BCL2L1-HRAS (B) and XRCC1-HRAS (D) are significantly co-mutated BRCA patients. Patients have mutations (Mutant [Mut] group) on both genes of BCL2L1-HRAS (C) or XRCC1-HRAS (E) are significantly correlate with poor survival rates comparing to wild-type [WT] group on both genes. P-value in B and D was computed by permutation set. P-value in C and E was computed by log-rank test. (PDF) [file pcbi.1007701.s006.pdf]

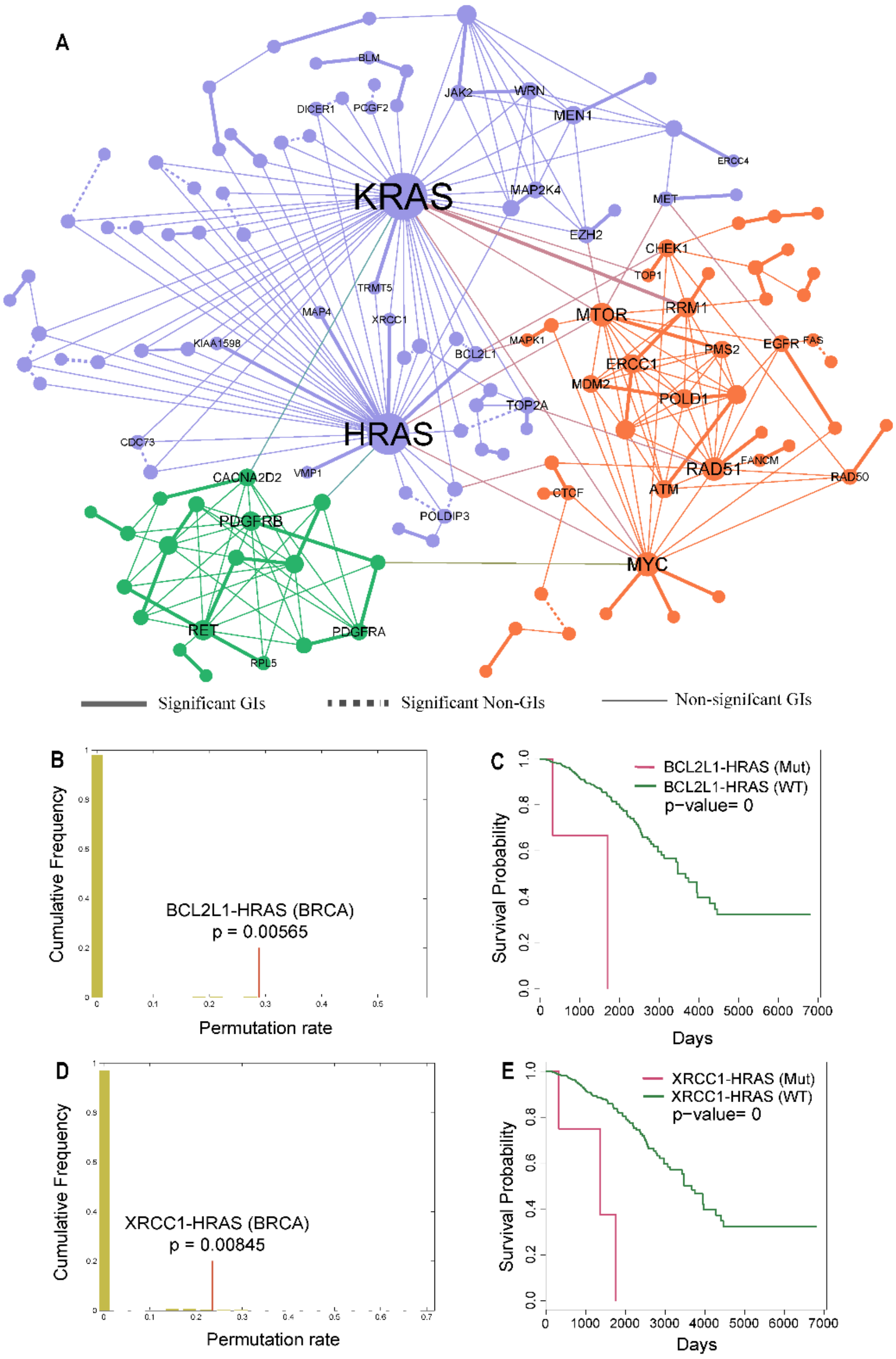

S6 Fig. INCM-predicted genetic interactions correlate with patient survivals in breast invasive carcinoma (BRCA). (**A**) The putative genetic interaction network in BRCA identified by individualized network-based co-mutation (INCM) measure. (**B**-**D**) The identified significantly putative genetic interactions correlate with patient survival rate. BCL2L1-HRAS (**B**) and XRCC1-HRAS (**D**) are significantly co-mutated BRCA patients. Patients have mutations (Mutant [Mut] group) on both genes of BCL2L1-HRAS (**C**) or XRCC1-HRAS (**E**) are significantly correlate with poor survival rates comparing to wild-type [WT] group on both genes. P-value in **B** and **D** was computed by permutation set. P-value in **C** and **E** was computed by logrank test.
