# Supplementary material for: Individualized genetic network analysis reveals new therapeutic vulnerabilities in 6,700 cancer genomes
Source: PLoS Comput Biol. 2020 Feb 26;16(2):e1007701. doi: 10.1371/journal.pcbi.1007701 (PMC7062285; doi:10.1371/journal.pcbi.1007701)
Supplement: S7 Fig — (PDF) [file pcbi.1007701.s007.pdf]

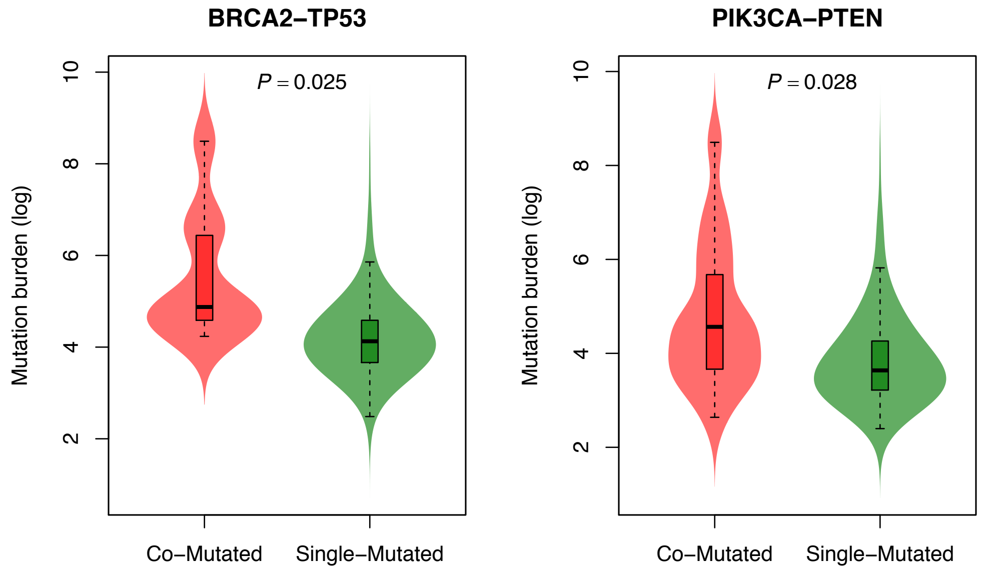

S7 Fig. Mutation burden for co-mutant (Co-mutated) tumors compared to single-mutant (Single-Mutated) tumors on BRCA2-TP53 and PIK3CA-PTEN in breast invasive carcinoma (BRCA).
