# Supplementary material for: Individualized genetic network analysis reveals new therapeutic vulnerabilities in 6,700 cancer genomes
Source: PLoS Comput Biol. 2020 Feb 26;16(2):e1007701. doi: 10.1371/journal.pcbi.1007701 (PMC7062285; doi:10.1371/journal.pcbi.1007701)
Supplement: S1 Table — (PDF) [file pcbi.1007701.s008.pdf]

S1 Table. The statistic of somatic mutation profiles across 14 cancer types collected from The Cancer Genome Atlas (TCGA) project. (PDF)

Cancer Types	Mutation profiles		
	# of Tumors	# of mutations	average # of mutations per tumor
BLCA	412	87,183	211.6
BRCA	1044	911,120	87.3
COAD	433	167,978	387.9
GBM	396	172,927	436.7
HNSC	510	21,128	132.7
KIRC	339	211,125	114.7
LAML	148	12,951	87.5
LUAD	569	143,683	252.5
LUSC	495	140,191	283.2
OV	443	63,988	144
PRAD	496	18,309	36.9
SKCM	468	239,606	511.9
THCA	494	11,817	23.9
UCEC	542	378,573	698.5
Pan-cancer	6789	2,580,579	380.1
