# Supplementary material for: Individualized genetic network analysis reveals new therapeutic vulnerabilities in 6,700 cancer genomes
Source: PLoS Comput Biol. 2020 Feb 26;16(2):e1007701. doi: 10.1371/journal.pcbi.1007701 (PMC7062285; doi:10.1371/journal.pcbi.1007701)
Supplement: S3 Table — (PDF) [file pcbi.1007701.s010.pdf]

S3 Table. The statistics of the cancer type-specifically co-expressed genetic networks for 14 cancer types. (PDF)

Cancer Types	Inferred genetic network	
	# of Genes	# of gene-gene interactions
BLCA	4,614	10,077
BRCA	4,989	12,550
COAD	4,672	10,298
GBM	4,213	9,017
HNSC	4,868	11,736
KIRC	4,852	12,924
LAML	4,234	9,837
LUAD	4,747	11,034
LUSC	4,746	10,551
OV	3,865	7,197
PRAD	4,792	12,999
SKCM	4,714	11,307
THCA	4,993	13,310
UCEC	4,036	8,434
Pan-cancer	5,907	19,846
