# Supplementary material for: Individualized genetic network analysis reveals new therapeutic vulnerabilities in 6,700 cancer genomes
Source: PLoS Comput Biol. 2020 Feb 26;16(2):e1007701. doi: 10.1371/journal.pcbi.1007701 (PMC7062285; doi:10.1371/journal.pcbi.1007701)
Supplement: S6 Table — The P values are calculated by the Wilcoxon rank-sum test over the corresponding gene set and human essential genes identified in cancer cell lines. (PDF) [file pcbi.1007701.s013.pdf]

S6 Table. Average cum*C* value for five functional gene sets. The P values are calculated by the Wilcoxon rank-sum test over the corresponding gene set and human essential genes identified in cancer cell lines. The standard deviation (±) was shown. (PDF)

Cancer Types	Driver Genes		CGC		CRF		DDR		Essential
	Ave cum*C*	P value	Ave cum*C*	P value	Ave cum*C*	P value	Ave cum*C*	P value	Ave cum*C*
BLCA	0.29 ±0.03	2.9e-10	0.23 ±0.02	1.7e-5	0.21 ±0.03	4.1e-3	0.25 ±0.06	2.6e-6	0.18 ±0.03
BRCA	0.16 ±0.02	2.2e-11	0.12 ±0.01	2.1e-5	0.12 ±0.02	2.0e-4	0.12 ±0.03	1.2e-4	0.09 ±0.02
COAD	0.31 ±0.04	5.4e-9	0.25 ±0.02	4.8e-11	0.25 ±0.03	1.6e-5	0.25 ±0.05	1.2e-9	0.17 ±0.03
GBM	0.28 ±0.03	1.4e-5	0.23 ±0.02	7.4e-7	0.23 ±0.03	0.024	0.24 ±0.05	6.4e-7	0.17 ±0.03
HNSC	0.12 ±0.02	1.0e-12	0.09 ±0.01	1.5e-10	0.07 ±0.01	5.1e-4	0.09 ±0.02	2.7e-7	0.05 ±0.01
KIRC	0.14 ±0.03	4.0e-8	0.10 ±0.02	1.3e-4	0.09 ±0.03	0.031	0.10 ±0.05	3.8e-3	0.07 ±0.03
LAML	0.19 ±0.09	1.3e-11	0.06 ±0.02	2.6e-3	0.04 ±0.02	0.312	0.05 ±0.04	0.015	0.04 ±0.03
LUAD	0.21 ±0.02	8.9e-10	0.19 ±0.02	2.5e-10	0.16 ±0.02	3.4e-3	0.18 ±0.04	1.0e-7	0.13 ±0.02
LUSC	0.22 ±0.03	4.3 e-8	0.20 ±0.02	1.3e-10	0.18 ±0.02	2.9e-4	0.19 ±0.04	9.2e-9	0.13 ±0.02
OV	0.20 ±0.05	8.4e-6	0.16 ±0.02	2.3e-5	0.16 ±0.03	1.2e-4	0.15 ±0.05	9.2e-4	0.11 ±0.03
PRAD	0.15 ±0.03	3.4e-17	0.08 ±0.02	3.3e-7	0.07 ±0.02	3.4e-4	0.07 ±0.03	0.0457	0.04 ±0.02
SKCM	0.19 ±0.02	1.1e-9	0.17 ±0.01	9.5e-9	0.16 ±0.02	6.1e-4	0.17 ±0.03	5.4e-8	0.12 ±0.02
THCA	0.21 ±0.10	4.3e-14	0.05 ±0.01	5.5e-6	0.04 ±0.02	3.3e-3	0.04 ±0.02	0.108	0.02 ±0.02
UCEC	0.30 ±0.04	2.8e-6	0.26 ±0.02	1.7e-5	0.25 ±0.03	0.033	0.25 ±0.05	1.3e-4	0.20 ±0.03
